# Modification of the Folmer primers for the cytochrome *c* oxidase gene facilitates identification of mosquitoes

**DOI:** 10.1186/s13071-022-05494-2

**Published:** 2022-11-22

**Authors:** Md Monirul Hoque, Matthew John Valentine, Patrick John Kelly, Subarna Barua, Daniel Felipe Barrantes Murillo, Chengming Wang

**Affiliations:** 1 College of Veterinary Medicine, College of Veterinary Medicine, Auburn University, Auburn, AL 36849-5519 USA; 2 School of Veterinary Medicine, Ross University, St. Kitts & Nevis, USA

**Keywords:** Folmer primers, Cytochrome *c* oxidase gene, Identification of mosquitoes, Morphological and molecular identification

## Abstract

**Background:**

Accurate identification of mosquito species is essential for the development and optimization of strategies to control mosquitoes and mosquito-borne diseases. Problems with the morphological identification of mosquito species have led to the use of molecular identification techniques, in particular the Folmer cytochrome *c* oxidase subunit I (*COI*) PCR system (FCOS), originally designed to identify a range of other invertebrates.

**Methods:**

As there can be difficulties identifying mosquitoes using FCOS, we re-evaluated the FCOS primers and developed a new *COI*-based SYBR PCR (the Auburn *COI* system—AUCOS) to improve the molecular identification of mosquitoes. Sequence data in GenBank for 33 species from 10 genera of mosquitoes were used to develop our AUCOS primers. Two molecular assays (AUCOS, FCOS) and morphological identification were carried out on mosquitoes collected from the field in Auburn, Alabama (USA) and on Saint Kitts.

**Results:**

With a convenience sample of individual mosquitoes comprising 19 species from six genera in Saint Kitts (*n* = 77) and Auburn (*n* = 48), our AUCOS provided higher-quality sequence data than FCOS. It also proved more sensitive than FCOS, successfully amplifying 67.5% (85/126) as opposed to 16.7% (21/126) of the samples. The species determined by morphology, or genus with damaged samples, matched that as determined by AUCOS for 84.9% (62/73) of the samples. Morphological classification was confirmed by FCOS with 81.0% (17/21) of samples producing utilizable sequences. While both FCOS and AUCOS correctly identified all the *Aedes*, *Anopheles*, *Deinocerites*, and *Uranotaenia* species in the study, identification of *Culex* species was less successful with both methods: 50.0% (3/6) by FCOS and 35.7% (5/14) by AUCOS.

**Conclusions:**

The AUCOS DNA barcoding system for mosquito species described in this study is superior to the existing FCOS for the identification of mosquito species. As AUCOS and FCOS amplify the same variable region of the *COI*, the large amount of existing data on GenBank can be used to identify mosquito species with sequences produced by either PCR.

**Graphical Abstract:**

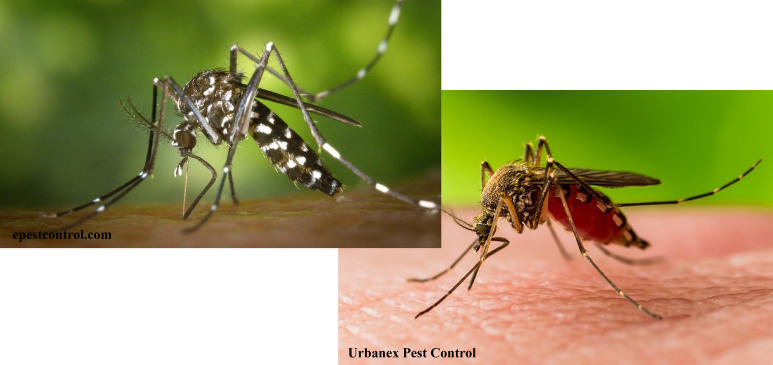

## Background

Mosquitoes are considered the most notorious vectors of pathogens among the hematophagous arthropods. More than half of the world’s people are at risk of exposure to pathogens borne by mosquitoes, principally those in the family Culicidae, which contains 42 genera and around 3560 species [1]. Members of the *Aedes*, *Anopheles*, and *Culex* genera are the most medically important species [2], transmitting a variety of viral pathogens including Zika, dengue, chikungunya, Japanese encephalitis, yellow fever, Rift Valley fever, West Nile, and western equine encephalitis viruses [3]. They also transmit parasites and protozoa such as *Dirofilaria* spp. and *Plasmodium* spp., respectively, and perhaps even bacteria, *Rickettsia felis* [4–6].

Despite decades of extensive research, there are considerable gaps in our current knowledge of the taxonomy of mosquitoes [7, 8]. This is of concern, as accurate identification of mosquito species is crucial for the development of optimal control programs and limiting the risk of mosquito-borne diseases [4]. This is becoming more important with expanding urbanization, agriculture, and tourism leading to increased numbers of people invading the natural habitats and breeding sites of mosquitoes [9–11].

External morphological features have conventionally been used as the gold standard for the identification of mosquito species, with unique anatomical features being utilized in taxonomic keys [12]. However, morphological identification requires experienced taxonomists, and the method is tedious and time-consuming. Further, damage to specimens during collection is common, and can lead to incomplete or misidentification of species [13]. A further limitation of morphological identification is that most taxonomic keys only apply to adult female mosquitoes and fourth-instar larvae because many morphological characteristics are not well developed in males and early larval stages [14, 15]. Further, morphological identification is complicated by phenotypic plasticity, genetic variation over time, and the presence of complexes of cryptic species which can differ in their capacity to transmit diseases [16].

The limitations of existing taxonomic keys based on morphology and recent advances in molecular methods have opened the possibility of using DNA sequences for species identification and the era of mosquito DNA barcoding [15, 17]. A hallmark publication by Folmer et al. [18] established “universal” DNA primers to amplify a fragment of the mitochondrial cytochrome *c* oxidase subunit I gene (*COI*) of invertebrates from 11 phyla. The *COI* contains highly conserved regions suitable for “universal primers” and regions with high sequence variation ideal for species identification [19]. The Barcode of Life Data System (BOLD) has selected this *COI* region as the “universal” or “Folmer” region and the standard marker for DNA barcoding [20]. Since Folmer et al.’s publication in 1994, nearly three million *COI* sequences have been uploaded to GenBank, and the Folmer *COI* polymerase chain reaction (PCR) system (FCOS) has become widely used for the identification of a variety of invertebrates, with over 15,000 citations (based on Google Scholar).

The FCOS universal primers have also been used for mosquito identification by many researchers [15, 21–32]. However, in our studies and those of others [5, 8, 29, 33–35], it has been noted that FCOS sometimes fails to adequately identify mosquito species. We thus re-examined the FCOS and developed primers better suited for mosquito identification.

## Methods

### Mosquitoes

Live adult mosquitoes were trapped in Auburn, Alabama, USA, with CO_2_-baited BG-2 Sentinel mosquito traps (BioQuip, Rancho Dominguez, CA, USA) and UV light-baited New Standard Miniature light traps (John W. Hock Company, Gainesville, FL, USA) as described previously [36, 37]. Live adult mosquitoes were also collected on Saint Kitts using the BG-2 Sentinel mosquito trap (BioQuip) baited with yeast-generated CO_2_ and BG-Sentinel lure (BioQuip) as described previously [38].

### Morphological identification of mosquitoes

The mosquitoes trapped in Auburn were identified in a chilled petri dish using a stereomicroscope (AmScope 3.5X-180X simul-focal stereo microscope, SM-4NTPZZ-144) and standard taxonomic keys [39, 40]. On Saint Kitts, the captured mosquitoes were identified using morphological keys [39–41] under a stereomicroscope with a chilled stage (Cole-Parmer, Vernon Hills, IL, USA) at 10–40× magnification [6, 38]. Due to difficulties in discriminating between sibling species [29, 42] of the Pipiens Assemblage [43], *Culex pipiens* that originated from Saint Kitts were designated *Cx. quinquefasciatus*, as this is the species previously reported on Saint Kitts [38, 44, 45]. After identification, the mosquitoes were stored at −80 °C until extraction of DNA for molecular testing.

### Washing and homogenization of the mosquitoes and DNA extraction

Mosquitoes representing the genera and species occurring most commonly in Auburn and Saint Kitts were selected for the study. Individual mosquitoes were placed in 2 ml microcentrifuge tubes, rinsed in 1× PBS, and left in 1 ml of 70% ethanol for 10 min. After discarding the ethanol, the mosquito was rinsed in fresh 1× PBS four times and homogenized with 400 µl of 1× PBS using three zirconia beads in a Precellys 24 lysis and homogenization instrument (Bertin Instruments, France) set at 5000 rpm for 15 s as described [36]. Homogenates were stored at −20 °C until DNA extraction using the High Pure PCR Template Preparation Kit (Roche Diagnostics, Indianapolis, IN, USA) as described previously [46, 47].

### Primer design and molecular identification of mosquitoes using the Auburn University *COI* PCR System (AUCOS) and FCOS

**Fig. 1:**
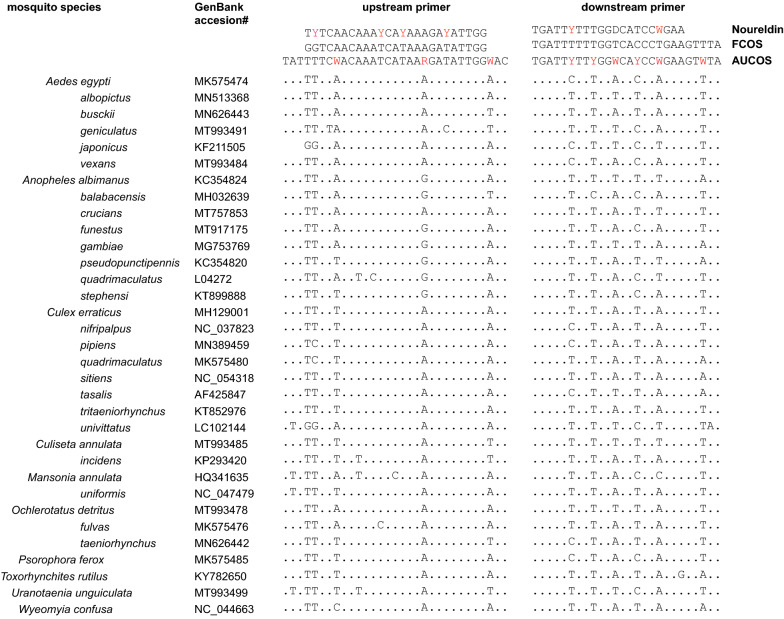
Alignment of primer targets and the *COI* nucleotide sequences of mosquito species. The primers are shown in the upper boxes, Noureldin’s primers on the first line, FCOS primers on the second, and AUCOS on the third. The upstream primers were used as shown, while the downstream primers were used as antisense oligonucleotides. Dots indicate nucleotides identical to both primers. Nine degenerate bases (in red font) were used in the primers (W represents A or T, R represents A or G, and Y represents C or T). The amplicon regions between the primers are highly polymorphic among different mosquito species (data not shown)

The *COI* sequences in GenBank (https://www.ncbi.nlm.nih.gov/nucleotide/) for medically important mosquito species and those seen in Saint Kitts and Auburn which had multiple *COI* sequences from different authors in GenBank were aligned using Clustal multiple sequence alignment in Vector NTI (InforMax Inc., North Bethesda, MD, USA). The alignments were used to identify conserved regions against which primers could be designed for the AUCOS (Fig. 1). The primers used for the FCOS were as described previously [18]. To address the nucleotide mismatches among mosquito species and develop the improved AUCOS primers, we incorporated three degenerate nucleotides in the FCOS upstream primer and six in the FCOS downstream primer (Fig. 1, Table 1). Because the first two nucleotides in the FCOS upstream primer (GG) were mismatched with most mosquito species we examined, for the AUCOS primer we used TT which occurred in most mosquito species. To improve the stringency, we also extended the numbers of bases in the forward (six base pairs [bp]) primer, as these were high across a wide range of mosquitoes.

**Table 1:** Primers used in this study targeting the mitochondrial *COI*

Primer	Sequences (5′ to 3′)	References
LCO 1490	5′-GGTCAACAAATCATAAAGATATTGG-3′	[17]
HCO 2198	5′-TAAACTTCAGGGTGACCAAAAAATCA-3′	
AU-COI-F	5′-TATTTTCWACAAATCATAARGATATTGGWAC-3′	This study
AU-COI-R	5′-TAWACTTCWGGRTGWCCRAARAATCA-3′	

The FCOS and AUCOS SYBR PCRs were performed in a LightCycler 480 II Thermocycler (Roche Diagnostics GmbH, Mannheim, Germany) using a high-stringency 18-cycle step-down temperature protocol as described previously [48]. In brief, for each PCR reaction, 10 µl of the extracted DNA was added to a 10 µl reaction mixture containing 5× PCR SYBR buffer, 400 µM dNTP (Roche Diagnostics GmbH), 0.34 units of Platinum Taq DNA Polymerase (Invitrogen, Carlsbad, CA, USA), 1 µM of each forward and reverse primer (Integrated DNA Technologies, Coralville, IA, USA) (Table 1), and a final volume of molecular-grade nuclease-free water. Thermal cycling consisted of 4 min incubation at 95 °C, 18 high-stringency step-down cycles followed by 30 relaxed-stringency fluorescence acquisition cycles. The 18 high-stringency step-down thermal cycles were 6 × 10 s at 95 °C, 10 s at 70 °C, 10 s at 72 °C; 9 × 10 s at 95 °C, 10 s at 68 °C, 10 s at 72 °C; 3 × 10 s at 95 °C, 10 s at 66 °C, 10 s at 72 °C. The relaxed-stringency fluorescence acquisition cycling consisted of 30 × 10 s at 95 °C, 10 s at 55 °C, and 30 s at 72 °C. PCR products were sent to ELIM Biopharmaceuticals (Hayward, CA, USA) for Sanger sequencing. To be included in the study, both forward and reverse DNA sequences were required which, after editing, trimming, and aligning (InforMax Inc., North Bethesda, MD, USA), produced contiguous sequences that could be compared to nucleotide sequences using the National Center for Biotechnology Information (NCBI) BLASTn database. Only database queries with 98% or greater similarity were considered valid molecular confirmation of a species and reported in our results [49, 50].

To determine the sensitivity of the AUCOS, positive PCR products from *Aedes japonicus*, *Anopheles punctipennis*, *Anopheles quadrimaculatus*, *Culex nigripalpus*, *Culex usquatissimus*, *Orthopodomyia alba*, *Psorophora ferox*, and *Uranotaenia sapphirina* were purified with the QIAquick PCR Purification Kit (QIAGEN, USA) according to the manufacturer’s protocol. The concentration of the purified DNA was measured using the Quant-iT™ PicoGreen ^®^ double-stranded (dsDNA) assay and the molarity of the DNA estimated using the calculated molecular mass of the amplicons. Dilutions were made to yield solution containing 10,000, 1000, 100, 10, and 1 gene copies/μl in T_10_E_0.1_ buffer, which were used as quantitative standards.

## Results

We used sequence data in GenBank for 33 species from 10 genera of mosquitoes to develop our AUCOS primers (Table 2; Fig. 1). Overall, there were 15 mismatches between the nucleotides in the FCOS primers (*n* = 51 nucleotides) and those in the corresponding sequences we studied in GenBank (Fig. 1). Of these, 10 occurred in every mosquito species we investigated. Ultimately, the AUCOS primers we developed contained 57 bp and amplified a 712-bp segment of the *COI* containing the highly polymorphic area that enabled the differentiation of mosquito species.

**Table 2:** Molecular identification of mosquitoes that were regarded as being reliably identified by their morphology

Mosquito sample	ID by morphology	ID by FCOS			ID by AUCOS		
		Mosquito	Nucleotide (%)	GenBank	Mosquito	Nucleotide (%)	GenBank
7	*Aedes aegypti*	NI			*Ae. aegypti*	321/325 (99%)	MN299002.1
AA1	*Ae. aegypti*	*Ae. aegypti*	681/685 (99%)	MK300221.1	*Ae. aegypti*	669/678 (99%)	MK300221.1
AA2	*Ae. aegypti*	*Ae. aegypti*	681/681 (100%)	MN298992.1	*Ae. aegypti*	675/678 (99%)	MN298993.1
AB	*Ae. busckii*	*Ae. busckii*	680/680 (100%)	MN626443.1	*Ae. busckii*	672/674 (99%)	MN626443.1
S166	*Ae. japonicus*	NI			*Ae. japonicus*	674/675 (99%)	KF211494.1
1	*Ae. taeniorhynchus*	NI			*Ae. taeniorhynchus*	680/681 (99%)	MN626442.1
2	*Ae. taeniorhynchus*	NI			*Ae. taeniorhynchus*	683/687 (99%)	MN626442.1
AT1	*Ae. tortilis*	*Ae. tortilis*	655/658 (99%)	JX259682.1	*Ae. tortilis*	654/657 (99%)	JX259682.1
AT2	*Ae. tortilis*	*Ae. tortilis*	657/658 (99%)	JX259682.1	*Ae. tortilis*	656/657 (99%)	JX259682.1
S203	*Anopheles crucians*	NI		MT040812.1	*An. crucians*	656/671 (98%)	MT040812.1
S171	*An. punctipennis*	*Ae. vexans*	678/684 (99%)	KP954638.1	*An. punctipennis*	655/657 (99%)	KR653634.100
S137	*An. punctipennis*	*An. punctipennis*	657/658 (99%)	KR666470.1	*An. punctipennis*	656/657 (99%)	KR666470.1
S198	*An. quadrimaculatus*	NI			*An. quadrimaculatus*	670/678 (99%)	L04272.1
S130	*Culex atratus*	NI			*Cx. pipiens*	702/709 (99%)	KP293422.1
S187	*Cx. coronator*	NI			*Cx. usquatus*	675/684 (99%)	NC_036005.1
S172	*Cx. coronator*	NI			*Cx. usquatissimus*	664/672 (99%)	NC_036007.1
S121	*Cx. erraticus*	*NI*			*Cx. pipiens*	704/710 (99%)	KP293422.1
S128	*Cx. erraticus*	NI			*Cx. pipiens*	694/698 (99%)	KP293425.1
S134	*Cx. nigripalpus*	*Cx. erraticus*	560/561 (99%)	MH128999.1	*Cx. erraticus*	665/673 (99%)	MH128999.1
S201	*Cx. nigripalpus*	*Cx. erraticus*	661/671 (99%)	MH128999.1	*Cx. erraticus*	663/672 (99%)	MH128999.1
S202	*Cx. quinquefasciatus*	*Cx. quinquefasciatus*	636/659 (100)	MW509603	*Cx. quinquefasciatus*	678/681 (99%)	KP293425.1
C1	*Cx. quinquefasciatus*	*Cx quinquefasciatus*	600/600 (100%)	MH463059.1	*Cx. quinquefasciatus*	684/687 (99%)	KP293425.1
C2	*Cx. pipiens*	*Cx. pipiens*	684/684 (100%)	MK714012.1	*Cx. pipiens*	707/713 (99%)	KP293422.1
S123	*Cx. quinquefasciatus*	NI			*Cx. quinquefasciatus*	674/677 (99%)	MK714001.1
10	*Cx. quinquefasciatus*	NI			*Cx. quinquefasciatus*	675/677 (99%)	KP293425.1
21	*Cx. tarsalis*	*Cx. coronator*	682/695 (98%)	NC_036006.1	*Cx. usquatissimus*	669/679 (99%)	NC_036007.1
S181	*Cx. tarsalis*	NI			*Cx. pipiens*	695/700 (99%)	KP293425.1
D1	*Deinocerites magnus*	*De. magnus*	678/684 (99%)	MH376751.1	*De. magnus*	672/681 (99%)	MH376751.1
D3	*De. magnus*	*De. magnus*	678/683 (99%)	MH376751.1	*De. magnus*	677/687 (99%)	MH376751.1
S199	*Psorophora howardii*	*Ps. howardii*	650/657 (99%)	MG242538.1	*Ps. howardii*	650/657 (99%)	MG242538.1
28	*Ps. pygmaea*	NI			*Ps. pygmaea*	657/657 (100%)	JX260116.1
P1	*Ps. pygmaea*	NI			*Ps. cingulata*	677/682 (99%)	KM592989.1
P3	*Ps. pygmaea*	*NI*			*Ps. cingulata*	675/680 (99%)	KM592989.1
S182	*Uranotaenia sapphirina*	*Ur. sapphirina*	637/639 (99%)	GU908127.1	*Ur. sapphirina*	638/639 (99%)	GU908127.1

*NI* not identifiable

The AUCOS proved more sensitive than FCOS on a convenience sample of 125 mosquitoes from Auburn (*n* = 48) and Saint Kitts (*n* = 77), providing high-quality sequence data for 67.5% (*n* = 85) as opposed to 16.7% (*n* = 21) of the samples, respectively. Thirty-four of the 85 mosquitoes which gave reliable AUCOS sequence data (Auburn = 24; Saint Kitts = 61) could be reliably identified morphologically to one of 19 species or species complexes from six genera (Table 2). The remainder (*n* = 51) were damaged and could only be identified by morphology to the genus level (*n* = 39) or as a mosquito (*n* = 12) (Table 3). Of the 85 samples with sequences by AUCOS which enabled species identification, only 24.7% (21/85) gave usable sequences for species identification with FCOS. Overall, the species determined by AUCOS and FCOS were the same as those determined by morphology for 67.6% (23/34) and 76.5% (13/17) of the samples, respectively. While all the *Aedes*, *Anopheles*, *Deinocerites*, and *Uranotaenia* species identified by AUCOS and FCOS corresponded with the species determined by morphology, only 35.7% (5/14) and 50.0% (3/6) of the *Culex* were identified correctly by AUCOS and FCOS, respectively. Two of the four *Psorophora* identified by AUCOS were the same as the species determined morphologically, while the single *Ps. howardii* was correctly identified by FCOS.

**Table 3:** Molecular identification of mosquitoes that gave usable sequences with AUCOS but could not be identified by their morphology or could only be identified to the genus level

Mosquito number	ID by morphology	ID by FCOS			ID by AUCOS		
		Mosquito	Nucleotide (%)	GenBank	Mosquito	Nucleotide (%)	GenBank
3	*Aedes* sp.	NI			*Aedes taeniorhynchus*	677/680 (99%)	MN626442.1
67	*Aedes* sp.	NI			*Ae. aegypti*	679/686 (99%)	MN298993.1
68	*Aedes* sp.	NI			*Ae. aegypti*	681/688 (99%)	MK300224.1
69	*Aedes* sp.	NI			*Ae. aegypti*	683/693 (99%)	MN298992.1
14	*Aedes* sp.	NI			*Ae. aegypti*	306/310 (99%)	MN299002.1
86	*Aedes* sp.	NI			*Ae. taeniorhynchus*	680/681 (99%)	MN626442.1
82	*Culex* sp.	NI			*Cx. quinquefasciatus*	676/678 (99%)	MK714012.1
S184	*Culex* sp.	NI			*Cx. erraticus*	665/673 (99%)	MH129001.1
S200	*Culex* sp.	NI			*Cx. pipiens*	680/685 (99%)	KP293425.1
8	*Culex* sp.	NI			*Cx. quinquefasciatus*	680/683 (99%)	KP293425.1
9	*Culex* sp.	NI			*Cx. quinquefasciatus*	680/683 (99%)	MK714012.1
15	*Culex* sp.	NI			*Cx. quinquefasciatus*	682/685 (99%)	KP293425.1
17	*Culex* sp.	NI			*Cx. quinquefasciatus*	684/691 (99%)	KP293422.1
18	*Culex* sp.	NI			*Cx. quinquefasciatus*	679/682 (99%)	MK714012.1
19	*Culex* sp.	NI			*Cx. quinquefasciatus*	678/682 (99%)	KP293422.1
20	*Culex* sp*.*	NI			*Cx. quinquefasciatus*	679/682 (99%)	KP293425.1
24	*Culex* sp.	NI			*Cx. quinquefasciatus*	676/678 (99%)	MK714012.1
31	*Culex* sp.	NI			*Cx. quinquefasciatus*	679/682 (99%)	KP293425.1
39	*Culex* sp.	NI			*Cx. quinquefasciatus*	684/691 (99%)	MK714012.1
43	*Culex* sp.	NI			*Cx. quinquefasciatus*	679/683 (99%)	KP293425.1
44	*Culex* sp.	NI			*Cx. quinquefasciatus*	677/679 (99%)	MK714012.1
45	*Culex* sp*.*	*Cx. quinquefasciatus*	678/679 (99%)	MK714012.1	*Cx. quinquefasciatus*	683/686 (99%)	KP293425.1
46	*Culex* sp.	NI			*Cx. quinquefasciatus*	687/691 (99%)	KP293425.1
47	*Culex* sp.	NI			*Cx. quinquefasciatus*	676/678 (99%)	MK714012.1
55	*Culex* sp*.*	NI			*Cx. quinquefasciatus*	682/685 (99%)	KP293425.1
58	*Culex* sp.	NI			*Cx. quinquefasciatus*	684/688 (99%)	KP293425.1
60	*Culex* sp.	NI			*Cx. quinquefasciatus*	702/713 (98%)	KP293422.1
61	*Culex* sp.	NI			*Cx. quinquefasciatus*	691/696 (99%)	KP293425.1
74	*Culex* sp.	NI			*Cx. quinquefasciatus*	694/700 (99%)	KP293422.1
84	*Culex* sp.	NI			*Cx. quinquefasciatus*	682/685 (99%)	KP293425.1
92	*Culex* sp.	NI			*Cx. quinquefasciatus*	683/687 (99%)	MN389462.1
93	*Culex* sp.	NI			*Cx. quinquefasciatus*	679/681 (99%)	KP293425.1
94	*Culex* sp.	NI			*Cx. quinquefasciatus*	702/713 (98%)	KP293422.1
99	*Culex* sp.	NI			*Cx. quinquefasciatus*	688/692 (99%)	KP293425.1
105	*Culex* sp*.*	NI			*Cx. quinquefasciatus*	683/687 (99%)	KP293425.1
107	*Culex* sp.	NI			*Cx. quinquefasciatus*	673/675 (99%)	MK714012.1
109	*Culex* sp*.*	NI			*Cx. quinquefasciatus*	673/675 (99%)	MK714012.1
S156	*Culex* sp.	*Cx. pipiens*	685/685 (100%)	MK714012.1	*Cx. pipiens*	675/677 (99%)	MK714012.1
S149	*Psorophora*	*Ps. confinnis*	653/658 (99%)	KY859921.1	*Ps. confinnis*	653/658 (99%)	KY859921.1
12	NI	NI			*Cx. quinquefasciatus*	645/651 (99%)	MN005046.1
57	NI	NI			*Cx. quinquefasciatus*	672/674 (99%)	MK714012.1
88	NI	NI			*Cx. quinquefasciatus*	689/701 (98%)	KP293425.1
70	NI	NI			*Cx. quinquefasciatus*	679/694 (98%)	KP293425.1
71	NI	NI			*Cx. quinquefasciatus*	682/686 (99%)	KP293425.1
72	NI	NI			*Cx. quinquefasciatus*	694/700 (99%)	KP293422.1
76	NI	NI			*Cx. quinquefasciatus*	684/687 (99%)	KP293425.1
96	NI	NI			*Cx. quinquefasciatus*	681/686 (99%)	KP293425.1
97	NI	NI			*Cx. quinquefasciatus*	684/687 (99%)	KP293425.1
S179	NI	NI			*Ae. triseriatus*	648/663 (98%)	MG242523.1
S183	NI	*Cx. nigripalpus*	675/679 (99%)	NC_037823.1	*Cx. nigripalpus*	663/674 (98%)	NC_037823.1
S197	NI	NI			*Ae. triseriatus*	654/665 (98%)	MG242523.1

*NI* not identifiable

The AUCOS also proved effective in confirming the genus of mosquito specimens that could only be identified by morphology to this level because of specimen damage (Table 3). All the *Aedes* (7) and *Culex* (32) and the one *Psorophora* were correctly identified. Further, in each case, the AUCOS gave the added benefit of providing not only genus but species data as well. For the 12 specimens so damaged that they could only be identified as mosquitoes, the AUCOS provided information on both the genus and species. The FCOS, however, only provided usable sequence data for four (8%) of the 51 mosquitoes identified only to genus level or just as a mosquito, although in each case indicating the same species as the AUCOS.

**Fig. 2:**
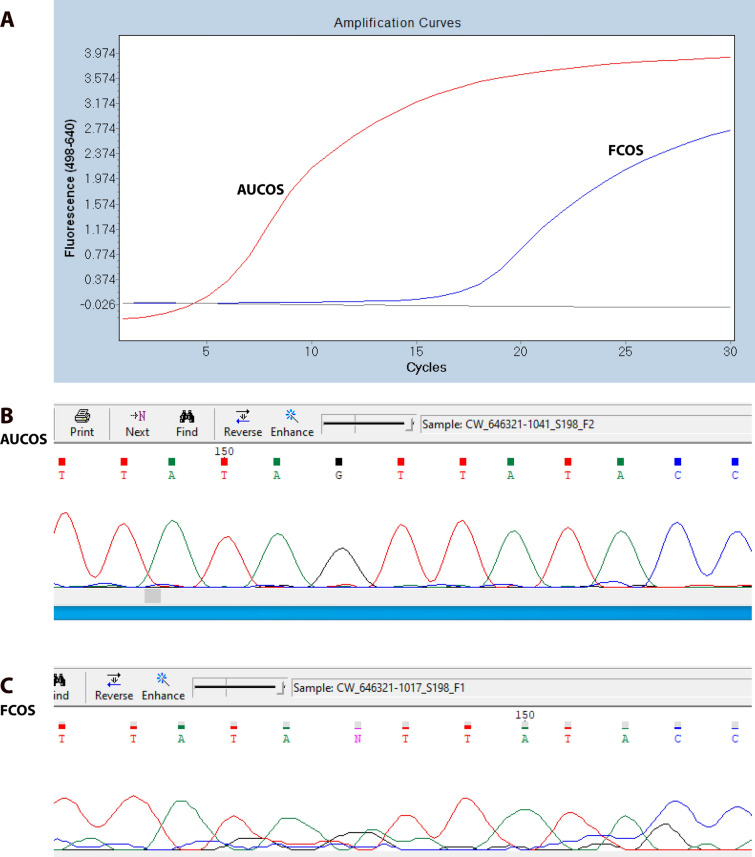
Amplification curves and Sanger sequences of the FCOS and AUCOS with DNA from an *Anopheles quadrimaculatus*. The Ct value with the FCOS is around 15.1, while that with the AUCOS is around 4.9 (**A**). The difference of 10 in Ct values indicates an approximately 1000-fold higher amplification efficiency in the AUCOS. The products of the AUCOS (**B**) provided higher-quality sequences than those of the FCOS (**C**)

In general, the DNA sequencing data from the AUCOS products was of higher quality than that from the FCOS products, with bases giving stronger and more distinct signals and thereby making nucleotide identification more reliable (Fig. 2). Also, AUCOS constantly gave higher copy numbers at lower cycle threshold (Ct) values than the FCOS for samples which were positive for both systems (Fig. 2). The AUCOS was also highly sensitive, detecting a single copy of *COI*/10 µl reaction for *Ae. japonicus*, *An. quadrimaculatus*, *Cx. nigripalpus*, *Cx. usquatissimus*, *Or. alba*, *Ps. ferox*, and *Ur. sapphirina*. It detected as few as 10 copies with *An. punctipennis*.

## Discussion

The cytochrome *c* oxidase subunit I is the largest of the mitochondrial-encoded cytochrome oxidase subunits and has highly conserved functional domains and variable regions [51]. It generally lacks indels, and changes in its amino acid sequence occur more slowly than those in any other mitochondrial gene, all aiding in the design of primers to be used in resolving deeper taxonomic affinities [15]. The Folmer universal primers developed for this gene have proven to be robust and have been used to discriminate many closely related invertebrate species in a variety of evolutionary studies and bio-identification systems [52].

To improve the existing FCOS primers for mosquitoes, we aligned *COI* sequences of 33 mosquito species from 10 genera that are medically important and/or can be found in our study area, Auburn and Saint Kitts. The alignments confirmed that the Folmer region of the *COI* is the most conserved and hence the most suitable fragment for the amplification and differentiation of mosquito species. There were, however, clear nucleotide mismatches between the FCOS primers and *COI* sequences of the 33 mosquito species we used to develop the AUCOS primers. Overall, 20% of the nucleotides in the FCOS primers did not match those of the reference mosquito sequences, which would be expected to lower target binding and PCR efficiency. With the extended length of the AUCOS primers we developed, the greater use of degenerate bases to replace mismatches, the high annealing temperatures, and the step-down thermal cycling we used, it was not unexpected that the AUCOS primers amplified a higher percentage of the mosquitoes we studied (67.5%; 85/126) than the FCOS primers (16.7%; 21/126). Species that were identified with AUCOS, but not FCOS, were *Ae. japonicus* (*n* = 1), *Ae. taeniorhynchus* (*n* = 2), *Ae. triseriatus* (*n* = 2), *An. quadrimaculatus* (*n* = 1)*, Cx. usquatissimus* (*n* = 2), *Cx. usquatus* (*n* = 1), *Ps. cingulata* (*n* = 1), and *Ps. pygmaea* (*n* = 1). This is presumably because of better alignment of the AUCOS primers with these species. The factors that influence primer binding are complex [53], and speculation upon which were at work in our study is beyond the scope of this article. It is of note, however, that the AUCOS primers we developed maintained their specificity, and we did not amplify non-target taxa, as has been described with FCOS [54]. This loss of specificity with FCOS is thought to result from the use of low annealing temperatures and multiple degenerate bases in the primers that are required to enable the system to amplify a very wide variety of invertebrates.

Our finding that not all mosquito DNA could be amplified with AUCOS has been reported previously with FCOS. The age of the specimens prior to extraction has been implicated, with museum and voucher specimens proving particularly difficult to amplify [29, 35]. This might have played a role in our study, as both Saint Kitts and Auburn have warm tropical climates that might lead to rapid desiccation, death, and DNA damage in mosquitoes trapped early in the 24-h trapping periods we used. It is of note that Noureldin et al. [35] used damp cotton pads in their traps and had very high success rates for amplifications with their PCRs using shortened and modified Folmer primers. Differences in DNA extraction methods were suggested to account for FCOS success rates varying from 43 to 85% by other workers [5]. In our study this did not appear to be the problem, as the same DNA extraction method was used throughout.

Overall, there was concordance in the use of morphology and both FCOS and AUCOS molecular methods (Table 3) in identifying mosquitoes from 10 species (*Ae. aegypti*, *Ae. busckii*, *Ae. tortilis*, *An. punctipennis*, *Cx. pipiens*, *Cx. quinquefasciatus*, *Deinocerites magnus*, *Ps. ferox*, *Ps. howardii*, and *Ur. sapphirina*). There were, however, discrepancies in the species identified by morphology and FCOS and AUCOS, particularly with *Culex* species (Table 3). This is not unexpected, as previous studies have also found that molecular techniques cannot reliably distinguish between *Culex* species [29, 42, 43, 55]. Also, morphological differentiation of *Culex* species is not always easy, even when performed by highly experienced taxonomists working with well-preserved specimens [56]. Our discordant results might, then, represent our inability to correctly identify *Culex* species based on morphology, or there might be incorrect GenBank entries as has recently been documented for *Culiseta* species, which are also difficult to differentiate by morphology [5]. Discordant results were also obtained with *Psorophora*; morphology indicated *Ps. pygmaea* while AUCOS identified *Ps. cingulata*. This discrepancy might have arisen because these species have only subtle differences in morphology, or perhaps observations were biased by the fact that only *Ps. pygmaea* has been recorded on Saint Kitts [38, 44, 45]. Further studies are underway in our laboratories to determine whether in fact both species are present on Saint Kitts.

Mosquitoes are very frail insects and thus frequently damaged in the traps that are used for surveillance studies. This greatly limits the data emanating from studies, as even minor damage can make morphological identification of the species difficult or impossible. Recent reports have shown that as many as 20% of captured mosquitoes might be damaged and 5% damaged so severely as to be unidentifiable [49, 57]. Our AUCOS proved to be very effective in determining the species of damaged mosquitoes where morphological criteria only enabled identification to the genus level, or where damage was so severe that even genus identification was not possible and they could only be recognized as mosquitoes.

## Conclusions

In summary, our studies have shown that AUCOS has distinct advantages over FCOS in identifying mosquitoes, in particular those species found in the Caribbean and southeastern USA where our study was conducted. By designing the AUCOS primers to more closely match the sequences of mosquito species reported in GenBank, the system provided a more sensitive method for producing high-quality *COI* sequences of medically important and other species of mosquitoes. The use of AUCOS would appear to be particularly valuable when attempts are made to identify specimens damaged during trapping, a common occurrence. As AUCOS and FCOS amplify the same variable region of the *COI*, the large amount of existing data on GenBank can be used to identify mosquito species with sequences produced by either PCR.

## Data Availability

The data that support the findings of this study are available from the corresponding author, CW, upon request.

## Abbreviations

*Ae.*: *Aedes*
*An.*: *Anopheles*
*Cx.*: *Culex*
*Oc.*: *Ochlerotatus*
*Ps.*: *Psorophora*
*Ur.*: *Uranotaenia*
*Wy.*: *Wyeomyia*
